# (20*S*)-Dammar-24-ene-3β,20-diol monohydrate from the bark of *Aglaia exima* (Meliaceae)

**DOI:** 10.1107/S1600536812034976

**Published:** 2012-08-15

**Authors:** Agus Safariari, Asep Supriadin, Unang Supratman, Khalijah Awang, Seik Weng Ng

**Affiliations:** a Department of Chemistry, Faculty of Mathematics and Natural Sciences, Padjadjaran University, Jatinangor 45363, West Java, Indonesia; b Department of Chemistry, University of Malaya, 50603 Kuala Lumpur, Malaysia; c Chemistry Department, Faculty of Science, King Abdulaziz University, PO Box 80203 Jeddah, Saudi Arabia

## Abstract

In the title compound {systematic name: (1*R*,2*R*,5*R*,7*R*,10*R*,11*R*,14*S*,15*R*)-14-[(2*S*)-2-hy­droxy-6-methyl­hept-5-en-2-yl]-2,6,6,10,11-penta­methyl­tetra­cyclo­[8.7.0.0^2,7^.0^11,15^]hepta­decan-5-ol monohydrate}, C_30_H_52_O_2_·H_2_O, the three fused cyclo­hexane rings adopt chair conformations and the hy­droxy substituent of one of these occupies an axial position. The fused cyclo­pentane ring adopts an envelope conformation (with the flap atom being the C atom bearing the methyl group) and the 3-methyl­but-2-enyl portion of its substituent is disordered over three sets of sites in a 0.413 (7):0.250 (7):0.337 (7) ratio. The O atoms of both water mol­ecules occupy special positions of 2 site symmetry. In the crystal, O_s_—H⋯O_w_ and O_w_—H⋯O_s_ (s = steroid and w = water) hydrogen bonds link hy­droxy groups and water mol­ecules, forming a three-dimensional network. The crystal studied was found to be a non-merohedral twin with a 0.518 (1):0.482 (1) component ratio.

## Related literature

For the isolation of 20*S*-dammar-24-ene-3*β*,20-diol from other plants, see: Anjaneyulu *et al.* (1985 ▶); Bianchini *et al.* (1988 ▶); Huang *et al.* (2010 ▶); Leonti *et al.* (2004 ▶); Pakhathirathien *et al.* (2005 ▶); Ukiya *et al.* (2010 ▶).
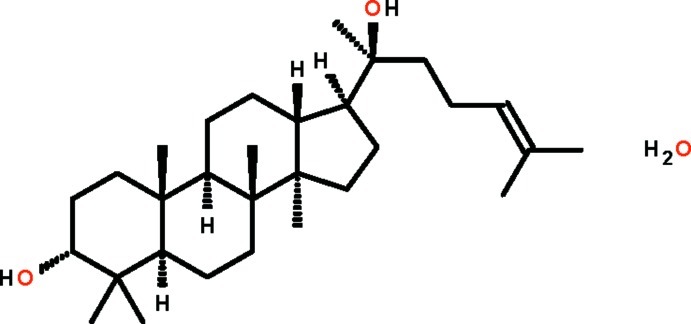


## Experimental

### 

#### Crystal data

C_30_H_52_O_2_·H_2_O
*M* *_r_* = 462.73
Tetragonal, 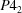

*a* = 19.9229 (1) Å
*c* = 7.3302 (1) Å
*V* = 2909.52 (4) Å^3^
*Z* = 4
Cu *K*α radiation
μ = 0.50 mm^−1^
*T* = 100 K
0.30 × 0.10 × 0.05 mm

#### Data collection

Agilent SuperNova Dual diffractometer with an Atlas detector
Absorption correction: multi-scan (*CrysAlis PRO*; Agilent, 2012 ▶) *T*_min_ = 0.864, *T*_max_ = 0.975
11893 measured reflections
15153 independent reflections
14049 reflections with *I* > 2σ(*I*)
*R*_int_ = 0.068

#### Refinement

*R*[*F* ^2^ > 2σ(*F* ^2^)] = 0.077
*wR*(*F* ^2^) = 0.266
*S* = 1.15
15153 reflections
341 parameters
45 restraints
H-atom parameters constrained
Δρ_max_ = 1.15 e Å^−3^
Δρ_min_ = −0.37 e Å^−3^
Absolute structure: Flack (1983 ▶), 2575 Friedel pairs
Flack parameter: 0.1 (3)

### 

Data collection: *CrysAlis PRO* (Agilent, 2012 ▶); cell refinement: *CrysAlis PRO*; data reduction: *CrysAlis PRO*; program(s) used to solve structure: *SHELXS97* (Sheldrick, 2008 ▶); program(s) used to refine structure: *SHELXL97* (Sheldrick, 2008 ▶); molecular graphics: *X-SEED* (Barbour, 2001 ▶); software used to prepare material for publication: *publCIF* (Westrip, 2010 ▶).

## Supplementary Material

Crystal structure: contains datablock(s) global, I. DOI: 10.1107/S1600536812034976/hb6931sup1.cif

Structure factors: contains datablock(s) I. DOI: 10.1107/S1600536812034976/hb6931Isup2.hkl

Supplementary material file. DOI: 10.1107/S1600536812034976/hb6931Isup3.cml

Additional supplementary materials:  crystallographic information; 3D view; checkCIF report

## Figures and Tables

**Table 1:** Hydrogen-bond geometry (Å, °)

*D*—H⋯*A*	*D*—H	H⋯*A*	*D*⋯*A*	*D*—H⋯*A*
O1—H1⋯O1w	0.84	1.96	2.745 (2)	154
O2—H2⋯O2w	0.84	2.03	2.809 (2)	154
O1w—H1w⋯O2^i^	0.84	1.88	2.712 (2)	171
O2w—H2w⋯O1^ii^	0.84	1.95	2.786 (2)	171

Symmetry codes: (i) 
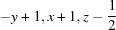
; (ii) 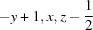.

**Fig. 1.**
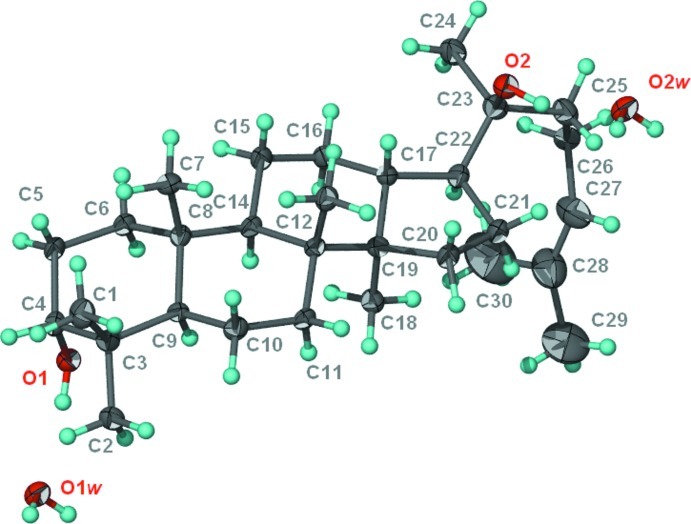
The molecular structure of the title compound at the 70% probability level; hydrogen atoms are drawn as spheres of arbitrary radius. The disorder in the 3-methylbut-2-enyl chain is not shown.

## supplementary crystallographic information

### Comment

The genus of *Aglaia* (Meliaceae family) comprises more than a hundred woody plant species, most of which are found in Indonesia. In this part of the world, the genus provides fruit and they are also used in traditional medicines. 20*S*-Dammar-24-ene-3*β*,20-diol has been isolated from several plants, *e.g.*, *Ceriops tagal* (Pakhathirathien *et al.*, 2005), *Ligustrum lucidum* (Huang *et al.*, 2010), *Olea madagascariensis* (Bianchini *et al.*, 1988), *Mangifera indica* (Anjaneyulu *et al.* (1985), *Mosquitoxylum jamaicense* (Anacardiaceae) (Leonti *et al.*, 2004) and *Shoreajavanica* (Dipterocarpaceae) (Ukiya *et al.*, 2010). The compound is also available commercially.

14-[(2*S*)-2-Hydroxy-6-methylhept-5-en-2-yl]-2,6,6,10,11-pentamethyltetracyclo[8.7.0^2,7^.0^11,15^]heptadecan-5-olcrystallizes as a monohydrate (Scheme I). The Flack parameter, calculated from 2574 Friedel pairs, is sufficiently well refined despite the somewhat large standard deviation. The few oxygen atoms together with twinning and disorder precluded a more accurate refinement; nevertheless, the absolute configuration is that expected from spectroscopic assignments.

The three cyclohexane rings that are fused together adopt chair conformations; the cyclopentane ring that is fused with a cyclohexane ring adopts an envelope conformation. The 3-methylbut-2-enyl portion of its substituent is disordered over three positions in an approximate 1:1:1 ratio. The O atoms of both water molecules lie on special positions of *2* site symmetry. Both hydroxy groups are hydrogen-bond donors to water molecules; the water molecules themselves are hydrogen-bond donors to hydroxy groups to generate a three-dimensional hydrogen-bonded network (Table 1).

### Experimental

*Aglaia exima* was collected from the Bogor Botanical Garden, West Java, Indonesia in July 2006. The plant was identified by Herbarium Bogoriense of Bogor city. The dried and milled bark (3 kg) was extracted succesively by *n*-hexane, ethyl acetate and methanol at room temperature. The ethyl acetate extract (300 g) was subjected to vacuum chromatography on silica gel G 60 by using a step gradient of *n*-hexane–ethyl acetate–methanol. The fraction eluted by *n*-hexane/ethyl acetate (3:2) was further separated by column chromatography on silica gel (chloroform: methanol; 9.5:0.5) to give a colorless solid (63 mg). Colourless prisms were obtained by recrystallization from ethyl acetate solution. The chemical structure was established by NMR spectroscopic analysis; however, the analysis did not note the presence of water.

### Refinement

Carbon- and oxygen-bound H-atoms were placed in calculated positions [C–H 0.98 to 1.00 Å, O–H 0.84 Å, *U*_iso_(H) 1.2 to 1.5*U*_eq_(C,*O*)] and were included in the refinement in the riding model approximation.

The 3-methylbut-2-enyl chain is disordered over three sites in a 0.413 (7): 0.250: 0.337 ratio. The temperature factors of the singly-primed and doubly-primed atoms were set to those of the unprimed ones; the anisotropic temperature factors were restrained to be nearly isotropic. The 1,2-related distances were restrained to within 0.01 Å.

The final difference Fourier map had a peak at 3.64 Å from H1*c*. The peak, which is close to a special position, could not be refined as a half oxygen atom.

The Flack parameter was calculated from 2574 Friedel pairs. The few oxygen atoms together with twinning and disorder precluded a more accurate refinement. The absolute configuration is that expected from spectroscopic assignments. Twinning and disorder probably contributed to the large weighting scheme.

The structure is a non-merohedral twin [twin law: -0.9998 - 0.0165 - 0.0977, 0.0160 - 0.9976 - 0.0931, -0.0130 - 0.0127) with a minor component of 48.2 (1)%.

### Crystal data

**Table:**

C_30_H_52_O_2_·H_2_O	*D*_x_ = 1.056 Mg m^−^^3^
*M**_r_* = 462.73	Cu *K*α radiation, λ = 1.54184 Å
Tetragonal, *P*4_2_	Cell parameters from 37417 reflections
Hall symbol: P 4c	θ = 3.1–76.4°
*a* = 19.9229 (1) Å	µ = 0.50 mm^−^^1^
*c* = 7.3302 (1) Å	*T* = 100 K
*V* = 2909.52 (4) Å^3^	Prism, colorless
*Z* = 4	0.30 × 0.10 × 0.05 mm
*F*(000) = 1032	

### Data collection

**Table:**

Agilent SuperNova Dual diffractometer with an Atlas detector	15153 independent reflections
Radiation source: SuperNova (Cu) X-ray Source	14049 reflections with *I* > 2σ(*I*)
Mirror monochromator	*R*_int_ = 0.068
Detector resolution: 10.4041 pixels mm^-1^	θ_max_ = 76.9°, θ_min_ = 3.1°
ω scan	*h* = −25→25
Absorption correction: multi-scan (*CrysAlis PRO*; Agilent, 2012)	*k* = −25→24
*T*_min_ = 0.864, *T*_max_ = 0.975	*l* = −9→7
11893 measured reflections	

### Refinement

**Table:**

Refinement on *F*^2^	Secondary atom site location: difference Fourier map
Least-squares matrix: full	Hydrogen site location: inferred from neighbouring sites
*R*[*F*^2^ > 2σ(*F*^2^)] = 0.077	H-atom parameters constrained
*wR*(*F*^2^) = 0.266	*w* = 1/[σ^2^(*F*_o_^2^) + (0.1593*P*)^2^ + 2.3284*P*] where *P* = (*F*_o_^2^ + 2*F*_c_^2^)/3
*S* = 1.15	(Δ/σ)_max_ = 0.001
15153 reflections	Δρ_max_ = 1.15 e Å^−^^3^
341 parameters	Δρ_min_ = −0.37 e Å^−^^3^
45 restraints	Absolute structure: Flack (1983), 2575 Friedel pairs
Primary atom site location: structure-invariant direct methods	Flack parameter: 0.1 (3)

### Fractional atomic coordinates and isotropic or equivalent isotropic displacement parameters (Å^2^)

**Table:**

	*x*	*y*	*z*	*U*_iso_*/*U*_eq_	Occ. (<1)
O1	0.40677 (8)	0.95002 (8)	0.4154 (3)	0.0199 (3)	
H1	0.4242	0.9693	0.3251	0.030*	
O2	0.08964 (8)	0.54803 (8)	0.4294 (3)	0.0208 (4)	
H2	0.0743	0.5323	0.3316	0.031*	
O1W	0.5000	1.0000	0.1754 (4)	0.0197 (5)	
H1W	0.4837	1.0303	0.1093	0.030*	
O2W	0.0000	0.5000	0.1640 (4)	0.0203 (5)	
H2W	0.0164	0.4694	0.0987	0.031*	
C1	0.23302 (12)	1.02383 (11)	0.3489 (4)	0.0233 (5)	
H1A	0.2503	1.0699	0.3528	0.035*	
H1B	0.2121	1.0129	0.4662	0.035*	
H1C	0.1996	1.0200	0.2513	0.035*	
C2	0.32326 (12)	0.99569 (11)	0.1298 (4)	0.0204 (5)	
H2A	0.3368	1.0429	0.1364	0.031*	
H2B	0.2905	0.9899	0.0314	0.031*	
H2C	0.3627	0.9677	0.1056	0.031*	
C3	0.29148 (12)	0.97463 (11)	0.3120 (3)	0.0177 (5)	
C4	0.34506 (11)	0.98337 (11)	0.4633 (4)	0.0184 (5)	
H4	0.3543	1.0323	0.4797	0.022*	
C5	0.32263 (12)	0.95406 (11)	0.6451 (3)	0.0195 (5)	
H5A	0.2830	0.9792	0.6899	0.023*	
H5B	0.3591	0.9594	0.7356	0.023*	
C6	0.30465 (11)	0.87956 (11)	0.6280 (3)	0.0179 (5)	
H6A	0.2899	0.8627	0.7486	0.022*	
H6B	0.3454	0.8542	0.5925	0.022*	
C7	0.18212 (12)	0.89138 (12)	0.5695 (4)	0.0201 (5)	
H7A	0.1854	0.9397	0.5926	0.030*	
H7B	0.1732	0.8679	0.6844	0.030*	
H7C	0.1455	0.8827	0.4835	0.030*	
C8	0.24895 (10)	0.86580 (11)	0.4874 (3)	0.0151 (4)	
C9	0.26992 (11)	0.89986 (11)	0.3032 (3)	0.0148 (4)	
H9	0.3115	0.8755	0.2656	0.018*	
C10	0.21932 (11)	0.88426 (11)	0.1525 (3)	0.0182 (5)	
H10A	0.2318	0.9088	0.0401	0.022*	
H10B	0.1741	0.8994	0.1904	0.022*	
C11	0.21803 (11)	0.80866 (11)	0.1139 (3)	0.0173 (4)	
H11A	0.2623	0.7950	0.0649	0.021*	
H11B	0.1839	0.7995	0.0188	0.021*	
C12	0.12674 (11)	0.77502 (12)	0.3267 (4)	0.0189 (5)	
H12A	0.0998	0.7600	0.2224	0.028*	
H12B	0.1176	0.8225	0.3508	0.028*	
H12C	0.1150	0.7484	0.4345	0.028*	
C13	0.20209 (11)	0.76580 (11)	0.2830 (3)	0.0149 (4)	
C14	0.24821 (11)	0.78826 (10)	0.4463 (3)	0.0151 (4)	
H14	0.2948	0.7782	0.4037	0.018*	
C15	0.23867 (12)	0.74306 (11)	0.6142 (4)	0.0196 (5)	
H15A	0.2701	0.7574	0.7112	0.024*	
H15B	0.1924	0.7487	0.6612	0.024*	
C16	0.25069 (11)	0.66882 (11)	0.5715 (3)	0.0181 (5)	
H16A	0.2981	0.6619	0.5357	0.022*	
H16B	0.2416	0.6414	0.6814	0.022*	
C17	0.20446 (11)	0.64684 (10)	0.4162 (3)	0.0166 (4)	
H17	0.1575	0.6572	0.4555	0.020*	
C18	0.29079 (11)	0.67911 (11)	0.1759 (4)	0.0197 (5)	
H18A	0.2984	0.6313	0.1526	0.030*	
H18B	0.3224	0.6949	0.2690	0.030*	
H18C	0.2976	0.7045	0.0630	0.030*	
C19	0.21845 (11)	0.68953 (11)	0.2437 (3)	0.0166 (4)	
C20	0.17282 (12)	0.65349 (11)	0.1054 (4)	0.0201 (5)	
H20A	0.1850	0.6657	−0.0213	0.024*	
H20B	0.1251	0.6648	0.1265	0.024*	
C21	0.18623 (12)	0.57823 (11)	0.1420 (4)	0.0212 (5)	
H21A	0.2237	0.5618	0.0653	0.025*	
H21B	0.1457	0.5512	0.1151	0.025*	
C22	0.20476 (11)	0.57331 (11)	0.3489 (3)	0.0172 (5)	
H22	0.2517	0.5559	0.3585	0.021*	
C23	0.15811 (11)	0.52502 (11)	0.4544 (4)	0.0199 (5)	
C24	0.17163 (13)	0.52639 (12)	0.6588 (4)	0.0247 (5)	
H24A	0.1409	0.4955	0.7207	0.037*	
H24B	0.1646	0.5720	0.7053	0.037*	
H24C	0.2181	0.5126	0.6821	0.037*	
C25	0.16250 (12)	0.45282 (11)	0.3808 (4)	0.0230 (5)	
H25A	0.1454	0.4528	0.2540	0.028*	0.413 (3)
H25B	0.1319	0.4244	0.4540	0.028*	0.413 (3)
H25C	0.1358	0.4504	0.2671	0.028*	0.250 (7)
H25D	0.1408	0.4227	0.4707	0.028*	0.250 (7)
H25E	0.1559	0.4542	0.2470	0.028*	0.337 (7)
H25F	0.1249	0.4266	0.4332	0.028*	0.337 (7)
C26	0.2328 (3)	0.4192 (5)	0.3806 (13)	0.0196 (19)	0.413 (3)
H26A	0.2592	0.4366	0.4850	0.024*	0.413 (3)
H26B	0.2274	0.3702	0.3973	0.024*	0.413 (3)
C27	0.2701 (7)	0.4317 (7)	0.2113 (18)	0.036 (2)	0.413 (3)
H27	0.2415	0.4194	0.1131	0.043*	0.413 (3)
C28	0.3265 (4)	0.4528 (5)	0.1393 (15)	0.0416 (18)	0.413 (3)
C29	0.3498 (5)	0.4646 (7)	−0.0560 (17)	0.059 (2)	0.413 (3)
H29A	0.3927	0.4417	−0.0759	0.089*	0.413 (3)
H29B	0.3554	0.5128	−0.0769	0.089*	0.413 (3)
H29C	0.3162	0.4468	−0.1409	0.089*	0.413 (3)
C30	0.3800 (6)	0.4710 (7)	0.277 (2)	0.074 (3)	0.413 (3)
H30A	0.3631	0.4623	0.4003	0.112*	0.413 (3)
H30B	0.3913	0.5186	0.2646	0.112*	0.413 (3)
H30C	0.4201	0.4437	0.2550	0.112*	0.413 (3)
C26'	0.2338 (6)	0.4245 (11)	0.340 (2)	0.0196 (19)	0.250 (7)
H26C	0.2632	0.4408	0.4395	0.024*	0.250 (7)
H26D	0.2305	0.3752	0.3550	0.024*	0.250 (7)
C27'	0.2706 (9)	0.4359 (15)	0.170 (2)	0.036 (2)	0.250 (7)
H27'	0.2516	0.4353	0.0509	0.043*	0.250 (7)
C28'	0.3390 (7)	0.4479 (8)	0.211 (3)	0.0416 (18)	0.250 (7)
C29'	0.3714 (9)	0.4563 (11)	0.024 (3)	0.059 (2)	0.250 (7)
H29D	0.3364	0.4576	−0.0696	0.089*	0.250 (7)
H29E	0.4016	0.4185	0.0007	0.089*	0.250 (7)
H29F	0.3970	0.4983	0.0215	0.089*	0.250 (7)
C30'	0.3828 (10)	0.4522 (13)	0.380 (4)	0.074 (3)	0.250 (7)
H30D	0.3546	0.4492	0.4889	0.112*	0.250 (7)
H30E	0.4068	0.4951	0.3799	0.112*	0.250 (7)
H30F	0.4152	0.4152	0.3792	0.112*	0.250 (7)
C26"	0.2285 (6)	0.4150 (10)	0.423 (2)	0.0196 (19)	0.337 (7)
H26E	0.2461	0.4296	0.5426	0.024*	0.337 (7)
H26F	0.2194	0.3662	0.4293	0.024*	0.337 (7)
C27"	0.2785 (6)	0.4279 (7)	0.2825 (19)	0.036 (2)	0.337 (7)
H27"	0.2648	0.4187	0.1609	0.043*	0.337 (7)
C28"	0.3442 (5)	0.4520 (5)	0.3021 (19)	0.0416 (18)	0.337 (7)
C29"	0.3880 (6)	0.4653 (7)	0.136 (2)	0.059 (2)	0.337 (7)
H29G	0.3598	0.4674	0.0270	0.089*	0.337 (7)
H29H	0.4208	0.4290	0.1234	0.089*	0.337 (7)
H29I	0.4116	0.5081	0.1522	0.089*	0.337 (7)
C30"	0.3691 (7)	0.4694 (9)	0.493 (2)	0.074 (3)	0.337 (7)
H30G	0.3606	0.5170	0.5174	0.112*	0.337 (7)
H30H	0.4174	0.4606	0.5005	0.112*	0.337 (7)
H30I	0.3454	0.4418	0.5827	0.112*	0.337 (7)

### Atomic displacement parameters (Å^2^)

**Table:**

	*U*^11^	*U*^22^	*U*^33^	*U*^12^	*U*^13^	*U*^23^
O1	0.0214 (8)	0.0221 (8)	0.0162 (9)	−0.0034 (6)	0.0000 (7)	−0.0006 (7)
O2	0.0198 (8)	0.0223 (8)	0.0202 (10)	−0.0020 (6)	0.0014 (7)	−0.0015 (7)
O1W	0.0230 (11)	0.0194 (11)	0.0168 (13)	0.0029 (8)	0.000	0.000
O2W	0.0254 (11)	0.0196 (11)	0.0161 (13)	0.0054 (8)	0.000	0.000
C1	0.0280 (12)	0.0164 (10)	0.0255 (14)	0.0030 (8)	−0.0015 (10)	−0.0008 (9)
C2	0.0240 (11)	0.0194 (10)	0.0179 (13)	−0.0032 (8)	0.0001 (9)	0.0031 (9)
C3	0.0223 (11)	0.0163 (10)	0.0145 (12)	−0.0017 (8)	0.0011 (9)	0.0016 (9)
C4	0.0227 (11)	0.0160 (10)	0.0164 (13)	−0.0021 (8)	−0.0011 (9)	−0.0029 (9)
C5	0.0273 (11)	0.0195 (10)	0.0116 (13)	−0.0055 (8)	−0.0018 (9)	−0.0019 (9)
C6	0.0234 (11)	0.0191 (10)	0.0113 (12)	−0.0031 (8)	−0.0019 (9)	0.0002 (9)
C7	0.0225 (11)	0.0215 (11)	0.0162 (13)	−0.0019 (8)	0.0048 (9)	−0.0021 (9)
C8	0.0170 (10)	0.0138 (10)	0.0145 (12)	0.0001 (7)	0.0002 (8)	−0.0013 (8)
C9	0.0181 (10)	0.0146 (9)	0.0118 (12)	−0.0011 (7)	0.0012 (8)	0.0009 (8)
C10	0.0231 (11)	0.0176 (10)	0.0140 (13)	0.0008 (8)	−0.0025 (9)	0.0018 (9)
C11	0.0230 (10)	0.0195 (10)	0.0093 (11)	−0.0023 (8)	−0.0023 (9)	0.0001 (9)
C12	0.0173 (10)	0.0219 (11)	0.0175 (13)	−0.0020 (8)	−0.0016 (9)	−0.0029 (9)
C13	0.0156 (10)	0.0179 (10)	0.0110 (11)	−0.0023 (7)	0.0000 (8)	−0.0007 (8)
C14	0.0162 (10)	0.0178 (10)	0.0112 (12)	0.0002 (7)	−0.0016 (8)	−0.0014 (9)
C15	0.0244 (11)	0.0200 (11)	0.0144 (13)	−0.0052 (8)	−0.0022 (9)	−0.0010 (9)
C16	0.0209 (11)	0.0187 (10)	0.0148 (12)	−0.0022 (8)	−0.0024 (9)	0.0025 (9)
C17	0.0191 (10)	0.0159 (10)	0.0149 (12)	−0.0034 (7)	0.0019 (9)	−0.0015 (9)
C18	0.0205 (10)	0.0170 (10)	0.0218 (13)	−0.0022 (8)	0.0035 (9)	−0.0019 (9)
C19	0.0175 (10)	0.0184 (11)	0.0140 (12)	−0.0026 (8)	0.0013 (9)	−0.0002 (9)
C20	0.0261 (11)	0.0200 (10)	0.0142 (12)	−0.0034 (8)	−0.0011 (9)	−0.0015 (9)
C21	0.0280 (12)	0.0197 (11)	0.0158 (13)	−0.0045 (8)	0.0011 (10)	−0.0030 (9)
C22	0.0194 (10)	0.0156 (10)	0.0167 (13)	0.0001 (7)	0.0004 (9)	−0.0020 (9)
C23	0.0203 (11)	0.0193 (11)	0.0200 (14)	−0.0015 (8)	−0.0002 (9)	0.0016 (9)
C24	0.0303 (12)	0.0227 (11)	0.0210 (14)	−0.0059 (9)	−0.0026 (11)	0.0041 (10)
C25	0.0251 (11)	0.0187 (11)	0.0253 (15)	−0.0039 (8)	−0.0015 (10)	0.0028 (10)
C26	0.0337 (17)	0.014 (3)	0.012 (6)	0.0011 (16)	−0.004 (3)	0.009 (4)
C27	0.034 (2)	0.021 (2)	0.053 (6)	0.0028 (16)	0.018 (3)	0.000 (5)
C28	0.028 (3)	0.041 (2)	0.057 (5)	−0.0006 (19)	0.002 (3)	−0.007 (4)
C29	0.029 (3)	0.075 (4)	0.073 (6)	−0.002 (3)	0.010 (3)	−0.002 (4)
C30	0.038 (3)	0.093 (5)	0.091 (7)	−0.014 (3)	−0.020 (5)	0.019 (5)
C26'	0.0337 (17)	0.014 (3)	0.012 (6)	0.0011 (16)	−0.004 (3)	0.009 (4)
C27'	0.034 (2)	0.021 (2)	0.053 (6)	0.0028 (16)	0.018 (3)	0.000 (5)
C28'	0.028 (3)	0.041 (2)	0.057 (5)	−0.0006 (19)	0.002 (3)	−0.007 (4)
C29'	0.029 (3)	0.075 (4)	0.073 (6)	−0.002 (3)	0.010 (3)	−0.002 (4)
C30'	0.038 (3)	0.093 (5)	0.091 (7)	−0.014 (3)	−0.020 (5)	0.019 (5)
C26"	0.0337 (17)	0.014 (3)	0.012 (6)	0.0011 (16)	−0.004 (3)	0.009 (4)
C27"	0.034 (2)	0.021 (2)	0.053 (6)	0.0028 (16)	0.018 (3)	0.000 (5)
C28"	0.028 (3)	0.041 (2)	0.057 (5)	−0.0006 (19)	0.002 (3)	−0.007 (4)
C29"	0.029 (3)	0.075 (4)	0.073 (6)	−0.002 (3)	0.010 (3)	−0.002 (4)
C30"	0.038 (3)	0.093 (5)	0.091 (7)	−0.014 (3)	−0.020 (5)	0.019 (5)

### Geometric parameters (Å, º)

**Table:**

O1—C4	1.441 (3)	C20—H20A	0.9900
O1—H1	0.8400	C20—H20B	0.9900
O2—C23	1.451 (3)	C21—C22	1.564 (4)
O2—H2	0.8400	C21—H21A	0.9900
O1W—H1W	0.8400	C21—H21B	0.9900
O2W—H2W	0.8400	C22—C23	1.545 (3)
C1—C3	1.546 (3)	C22—H22	1.0000
C1—H1A	0.9800	C23—C24	1.523 (4)
C1—H1B	0.9800	C23—C25	1.539 (3)
C1—H1C	0.9800	C24—H24A	0.9800
C2—C3	1.536 (3)	C24—H24B	0.9800
C2—H2A	0.9800	C24—H24C	0.9800
C2—H2B	0.9800	C25—C26"	1.547 (7)
C2—H2C	0.9800	C25—C26	1.553 (6)
C3—C4	1.549 (3)	C25—C26'	1.557 (8)
C3—C9	1.552 (3)	C25—H25A	0.9900
C4—C5	1.522 (3)	C25—H25B	0.9900
C4—H4	1.0000	C25—H25C	0.9900
C5—C6	1.532 (3)	C25—H25D	0.9902
C5—H5A	0.9900	C25—H25E	0.9900
C5—H5B	0.9900	C25—H25F	0.9901
C6—C8	1.539 (3)	C26—C27	1.467 (8)
C6—H6A	0.9900	C26—H26A	0.9900
C6—H6B	0.9900	C26—H26B	0.9900
C7—C8	1.547 (3)	C27—C28	1.311 (13)
C7—H7A	0.9800	C27—H27	0.9500
C7—H7B	0.9800	C28—C30	1.510 (14)
C7—H7C	0.9800	C28—C29	1.523 (13)
C8—C9	1.567 (3)	C29—H29A	0.9800
C8—C14	1.574 (3)	C29—H29B	0.9800
C9—C10	1.528 (3)	C29—H29C	0.9800
C9—H9	1.0000	C30—H30A	0.9800
C10—C11	1.533 (3)	C30—H30B	0.9800
C10—H10A	0.9900	C30—H30C	0.9800
C10—H10B	0.9900	C26'—C27'	1.465 (10)
C11—C13	1.539 (3)	C26'—H26C	0.9900
C11—H11A	0.9900	C26'—H26D	0.9900
C11—H11B	0.9900	C27'—C28'	1.417 (16)
C12—C13	1.546 (3)	C27'—H27'	0.9500
C12—H12A	0.9800	C28'—C30'	1.514 (16)
C12—H12B	0.9800	C28'—C29'	1.523 (14)
C12—H12C	0.9800	C29'—H29D	0.9800
C13—C14	1.574 (3)	C29'—H29E	0.9800
C13—C19	1.581 (3)	C29'—H29F	0.9800
C14—C15	1.537 (3)	C30'—H30D	0.9800
C14—H14	1.0000	C30'—H30E	0.9800
C15—C16	1.531 (3)	C30'—H30F	0.9800
C15—H15A	0.9900	C26"—C27"	1.454 (9)
C15—H15B	0.9900	C26"—H26E	0.9900
C16—C17	1.528 (3)	C26"—H26F	0.9900
C16—H16A	0.9900	C27"—C28"	1.401 (14)
C16—H16B	0.9900	C27"—H27"	0.9500
C17—C22	1.546 (3)	C28"—C29"	1.518 (14)
C17—C19	1.549 (3)	C28"—C30"	1.522 (15)
C17—H17	1.0000	C29"—H29G	0.9800
C18—C19	1.538 (3)	C29"—H29H	0.9800
C18—H18A	0.9800	C29"—H29I	0.9800
C18—H18B	0.9800	C30"—H30G	0.9800
C18—H18C	0.9800	C30"—H30H	0.9800
C19—C20	1.540 (3)	C30"—H30I	0.9800
C20—C21	1.547 (3)		
			
C4—O1—H1	109.5	H18A—C18—H18B	109.5
C23—O2—H2	109.5	C19—C18—H18C	109.5
C3—C1—H1A	109.5	H18A—C18—H18C	109.5
C3—C1—H1B	109.5	H18B—C18—H18C	109.5
H1A—C1—H1B	109.5	C18—C19—C20	106.1 (2)
C3—C1—H1C	109.5	C18—C19—C17	110.99 (19)
H1A—C1—H1C	109.5	C20—C19—C17	100.09 (18)
H1B—C1—H1C	109.5	C18—C19—C13	112.45 (17)
C3—C2—H2A	109.5	C20—C19—C13	116.53 (19)
C3—C2—H2B	109.5	C17—C19—C13	109.99 (19)
H2A—C2—H2B	109.5	C19—C20—C21	103.65 (19)
C3—C2—H2C	109.5	C19—C20—H20A	111.0
H2A—C2—H2C	109.5	C21—C20—H20A	111.0
H2B—C2—H2C	109.5	C19—C20—H20B	111.0
C2—C3—C1	106.84 (19)	C21—C20—H20B	111.0
C2—C3—C4	107.92 (19)	H20A—C20—H20B	109.0
C1—C3—C4	108.8 (2)	C20—C21—C22	105.65 (19)
C2—C3—C9	109.91 (19)	C20—C21—H21A	110.6
C1—C3—C9	114.03 (19)	C22—C21—H21A	110.6
C4—C3—C9	109.15 (18)	C20—C21—H21B	110.6
O1—C4—C5	106.67 (19)	C22—C21—H21B	110.6
O1—C4—C3	111.20 (19)	H21A—C21—H21B	108.7
C5—C4—C3	112.43 (18)	C23—C22—C17	115.33 (19)
O1—C4—H4	108.8	C23—C22—C21	112.48 (19)
C5—C4—H4	108.8	C17—C22—C21	104.44 (18)
C3—C4—H4	108.8	C23—C22—H22	108.1
C4—C5—C6	111.6 (2)	C17—C22—H22	108.1
C4—C5—H5A	109.3	C21—C22—H22	108.1
C6—C5—H5A	109.3	O2—C23—C24	106.6 (2)
C4—C5—H5B	109.3	O2—C23—C25	107.73 (18)
C6—C5—H5B	109.3	C24—C23—C25	110.6 (2)
H5A—C5—H5B	108.0	O2—C23—C22	107.80 (18)
C5—C6—C8	113.33 (19)	C24—C23—C22	112.02 (19)
C5—C6—H6A	108.9	C25—C23—C22	111.9 (2)
C8—C6—H6A	108.9	C23—C24—H24A	109.5
C5—C6—H6B	108.9	C23—C24—H24B	109.5
C8—C6—H6B	108.9	H24A—C24—H24B	109.5
H6A—C6—H6B	107.7	C23—C24—H24C	109.5
C8—C7—H7A	109.5	H24A—C24—H24C	109.5
C8—C7—H7B	109.5	H24B—C24—H24C	109.5
H7A—C7—H7B	109.5	C23—C25—C26"	115.7 (9)
C8—C7—H7C	109.5	C23—C25—C26	117.0 (5)
H7A—C7—H7C	109.5	C23—C25—C26'	117.3 (10)
H7B—C7—H7C	109.5	C23—C25—H25A	108.0
C6—C8—C7	107.5 (2)	C26—C25—H25A	108.0
C6—C8—C9	107.90 (17)	C23—C25—H25B	108.0
C7—C8—C9	114.95 (18)	C26—C25—H25B	108.0
C6—C8—C14	108.04 (17)	H25A—C25—H25B	107.3
C7—C8—C14	112.92 (17)	C23—C25—H25C	108.1
C9—C8—C14	105.23 (18)	C26—C25—H25C	117.6
C10—C9—C3	114.06 (18)	C26'—C25—H25C	108.1
C10—C9—C8	111.03 (18)	C23—C25—H25D	107.9
C3—C9—C8	117.00 (19)	C23—C25—H25E	108.4
C10—C9—H9	104.4	C26"—C25—H25E	108.8
C3—C9—H9	104.4	C23—C25—H25F	108.3
C8—C9—H9	104.4	C26"—C25—H25F	108.0
C9—C10—C11	110.16 (18)	H25E—C25—H25F	107.4
C9—C10—H10A	109.6	C27—C26—C25	112.6 (7)
C11—C10—H10A	109.6	C27—C26—H26A	109.1
C9—C10—H10B	109.6	C25—C26—H26A	109.1
C11—C10—H10B	109.6	C27—C26—H26B	109.1
H10A—C10—H10B	108.1	C25—C26—H26B	109.1
C10—C11—C13	113.6 (2)	H26A—C26—H26B	107.8
C10—C11—H11A	108.9	C28—C27—C26	145.9 (14)
C13—C11—H11A	108.9	C28—C27—H27	107.0
C10—C11—H11B	108.9	C26—C27—H27	107.0
C13—C11—H11B	108.9	C27—C28—C30	114.4 (12)
H11A—C11—H11B	107.7	C27—C28—C29	133.5 (11)
C13—C12—H12A	109.5	C30—C28—C29	112.1 (9)
C13—C12—H12B	109.5	C27'—C26'—C25	124.3 (14)
H12A—C12—H12B	109.5	C27'—C26'—H26C	106.2
C13—C12—H12C	109.5	C25—C26'—H26C	106.2
H12A—C12—H12C	109.5	C27'—C26'—H26D	106.2
H12B—C12—H12C	109.5	C25—C26'—H26D	106.2
C11—C13—C12	107.53 (19)	H26C—C26'—H26D	106.4
C11—C13—C14	109.55 (17)	C28'—C27'—C26'	109.0 (16)
C12—C13—C14	112.08 (19)	C28'—C27'—H27'	125.5
C11—C13—C19	110.12 (19)	C26'—C27'—H27'	125.5
C12—C13—C19	110.62 (17)	C27'—C28'—C30'	137.5 (17)
C14—C13—C19	106.95 (17)	C27'—C28'—C29'	103.5 (14)
C15—C14—C13	111.72 (17)	C30'—C28'—C29'	118.9 (15)
C15—C14—C8	115.04 (19)	C28'—C29'—H29D	109.5
C13—C14—C8	115.47 (18)	C28'—C29'—H29E	109.5
C15—C14—H14	104.3	H29D—C29'—H29E	109.5
C13—C14—H14	104.3	C28'—C29'—H29F	109.5
C8—C14—H14	104.3	H29D—C29'—H29F	109.5
C16—C15—C14	112.5 (2)	H29E—C29'—H29F	109.5
C16—C15—H15A	109.1	C28'—C30'—H30D	109.5
C14—C15—H15A	109.1	C28'—C30'—H30E	109.5
C16—C15—H15B	109.1	H30D—C30'—H30E	109.5
C14—C15—H15B	109.1	C28'—C30'—H30F	109.5
H15A—C15—H15B	107.8	H30D—C30'—H30F	109.5
C17—C16—C15	109.57 (18)	H30E—C30'—H30F	109.5
C17—C16—H16A	109.8	C27"—C26"—C25	110.9 (9)
C15—C16—H16A	109.8	C27"—C26"—H26E	109.5
C17—C16—H16B	109.8	C25—C26"—H26E	109.5
C15—C16—H16B	109.8	C27"—C26"—H26F	109.5
H16A—C16—H16B	108.2	C25—C26"—H26F	109.5
C16—C17—C22	120.46 (19)	H26E—C26"—H26F	108.1
C16—C17—C19	110.00 (17)	C28"—C27"—C26"	128.9 (14)
C22—C17—C19	105.02 (19)	C28"—C27"—H27"	115.5
C16—C17—H17	106.9	C26"—C27"—H27"	115.5
C22—C17—H17	106.9	C27"—C28"—C29"	121.0 (11)
C19—C17—H17	106.9	C27"—C28"—C30"	118.5 (11)
C19—C18—H18A	109.5	C29"—C28"—C30"	120.4 (11)
C19—C18—H18B	109.5		
			
C2—C3—C4—O1	−51.8 (2)	C11—C13—C19—C18	54.0 (3)
C1—C3—C4—O1	−167.36 (18)	C12—C13—C19—C18	172.7 (2)
C9—C3—C4—O1	67.6 (2)	C14—C13—C19—C18	−65.0 (2)
C2—C3—C4—C5	−171.33 (18)	C11—C13—C19—C20	−68.8 (2)
C1—C3—C4—C5	73.1 (2)	C12—C13—C19—C20	49.9 (3)
C9—C3—C4—C5	−51.9 (2)	C14—C13—C19—C20	172.23 (19)
O1—C4—C5—C6	−65.2 (2)	C11—C13—C19—C17	178.24 (18)
C3—C4—C5—C6	56.9 (3)	C12—C13—C19—C17	−63.0 (2)
C4—C5—C6—C8	−58.0 (3)	C14—C13—C19—C17	59.3 (2)
C5—C6—C8—C7	−72.1 (2)	C18—C19—C20—C21	71.1 (2)
C5—C6—C8—C9	52.4 (2)	C17—C19—C20—C21	−44.3 (2)
C5—C6—C8—C14	165.72 (19)	C13—C19—C20—C21	−162.83 (19)
C2—C3—C9—C10	−59.2 (2)	C19—C20—C21—C22	29.7 (2)
C1—C3—C9—C10	60.8 (3)	C16—C17—C22—C23	86.4 (3)
C4—C3—C9—C10	−177.36 (19)	C19—C17—C22—C23	−148.94 (19)
C2—C3—C9—C8	168.87 (18)	C16—C17—C22—C21	−149.6 (2)
C1—C3—C9—C8	−71.2 (3)	C19—C17—C22—C21	−25.0 (2)
C4—C3—C9—C8	50.7 (2)	C20—C21—C22—C23	122.9 (2)
C6—C8—C9—C10	176.04 (17)	C20—C21—C22—C17	−2.9 (2)
C7—C8—C9—C10	−64.0 (2)	C17—C22—C23—O2	62.8 (3)
C14—C8—C9—C10	60.9 (2)	C21—C22—C23—O2	−56.9 (2)
C6—C8—C9—C3	−50.6 (2)	C17—C22—C23—C24	−54.1 (3)
C7—C8—C9—C3	69.3 (2)	C21—C22—C23—C24	−173.8 (2)
C14—C8—C9—C3	−165.81 (18)	C17—C22—C23—C25	−179.0 (2)
C3—C9—C10—C11	161.98 (19)	C21—C22—C23—C25	61.4 (2)
C8—C9—C10—C11	−63.3 (2)	O2—C23—C25—C26"	−170.6 (5)
C9—C10—C11—C13	56.8 (2)	C24—C23—C25—C26"	−54.5 (6)
C10—C11—C13—C12	72.8 (2)	C22—C23—C25—C26"	71.1 (6)
C10—C11—C13—C14	−49.3 (2)	O2—C23—C25—C26	175.8 (4)
C10—C11—C13—C19	−166.64 (17)	C24—C23—C25—C26	−68.1 (4)
C11—C13—C14—C15	−175.11 (18)	C22—C23—C25—C26	57.5 (5)
C12—C13—C14—C15	65.6 (2)	O2—C23—C25—C26'	162.7 (7)
C19—C13—C14—C15	−55.8 (2)	C24—C23—C25—C26'	−81.2 (7)
C11—C13—C14—C8	50.9 (2)	C22—C23—C25—C26'	44.4 (7)
C12—C13—C14—C8	−68.4 (2)	C23—C25—C26—C27	−89.5 (10)
C19—C13—C14—C8	170.25 (18)	C26"—C25—C26—C27	−176 (6)
C6—C8—C14—C15	56.6 (2)	C26'—C25—C26—C27	5 (5)
C7—C8—C14—C15	−62.2 (3)	C25—C26—C27—C28	127.9 (19)
C9—C8—C14—C15	171.71 (18)	C26—C27—C28—C30	3 (3)
C6—C8—C14—C13	−170.91 (19)	C26—C27—C28—C29	−175.1 (16)
C7—C8—C14—C13	70.3 (3)	C23—C25—C26'—C27'	−84 (2)
C9—C8—C14—C13	−55.8 (2)	C26"—C25—C26'—C27'	−177 (6)
C13—C14—C15—C16	55.9 (2)	C26—C25—C26'—C27'	−176 (7)
C8—C14—C15—C16	−169.89 (17)	C25—C26'—C27'—C28'	139 (2)
C14—C15—C16—C17	−56.4 (2)	C26'—C27'—C28'—C30'	0 (4)
C15—C16—C17—C22	−178.3 (2)	C26'—C27'—C28'—C29'	178.1 (19)
C15—C16—C17—C19	59.5 (2)	C23—C25—C26"—C27"	−87.7 (15)
C16—C17—C19—C18	62.3 (2)	C26—C25—C26"—C27"	11 (4)
C22—C17—C19—C18	−68.7 (2)	C26'—C25—C26"—C27"	12 (3)
C16—C17—C19—C20	174.03 (18)	C25—C26"—C27"—C28"	125.6 (15)
C22—C17—C19—C20	43.0 (2)	C26"—C27"—C28"—C29"	−177.1 (14)
C16—C17—C19—C13	−62.8 (2)	C26"—C27"—C28"—C30"	−1 (2)
C22—C17—C19—C13	166.21 (17)		

### Hydrogen-bond geometry (Å, º)

**Table:**

*D*—H···*A*	*D*—H	H···*A*	*D*···*A*	*D*—H···*A*
O1—H1···O1w	0.84	1.96	2.745 (2)	154
O2—H2···O2w	0.84	2.03	2.809 (2)	154
O1w—H1w···O2^i^	0.84	1.88	2.712 (2)	171
O2w—H2w···O1^ii^	0.84	1.95	2.786 (2)	171

Symmetry codes: (i) −*y*+1, *x*+1, *z*−1/2; (ii) −*y*+1, *x*, *z*−1/2.
